# Digital Patient Education on Xanthelasma Palpebrarum: A Content Analysis

**DOI:** 10.3390/clinpract13050108

**Published:** 2023-09-29

**Authors:** Kevin J. Varghese, Som P. Singh, Fahad M. Qureshi, Shreevarsha Shreekumar, Aarya Ramprasad, Fawad Qureshi

**Affiliations:** 1 Department of Biomedical Sciences, University of Missouri Kansas City School of Medicine, Kansas City, MO 64108, USA; fq7mc@mail.umkc.edu (F.M.Q.); ss5br@mail.umkc.edu (S.S.); aarya.ramprasad@mail.umkc.edu (A.R.); 2 Department of Nephrology, Mayo Clinic Alix School of Medicine, Rochester, MN 55905, USA; qureshi.fawad@mayo.edu

**Keywords:** xanthelasma palpebrarum, patient education, digital health, readability, content analysis

## Abstract

Patient education has been transformed using digital media and online repositories which disseminate information with greater efficiency. In dermatology, this transformation has allowed for patients to gain education on common cutaneous conditions and improve health literacy. Xanthelasma palpebrarum is one of the most common cutaneous conditions, yet there is a poor understanding of how digital materials affect health literacy on this condition. Our study aimed to address this paucity of literature utilizing Brief DISCERN, Rothwell’s Classification of Questions, and six readability calculations. The findings of this study indicate a poor-quality profile (Brief DISCERN < 16) regarding digital materials and readability scores which do not meet grade-level recommendations in the United States. This indicates a need to improve the current body of educational materials used by clinicians for diagnosing and managing xanthelasma palpebrarum.

## 1. Introduction

The utilization of digital applications in healthcare serves an imperative role in the physician–patient relationship [1]. Mobile health applications, telehealth, and multiple internet-based resources allow for the efficient dissemination of information and serve as a primary medium for healthcare literacy [2,3,4]. Moreover, patient education and healthcare literacy are closely tied to patient outcomes. Poor patient education and health literacy are associated with suboptimal healthcare outcomes [5,6]. Among dermatologic conditions, there is a growing emphasis on improving patient education for the goal of improving healthcare outcomes [7].

Xanthomas are common well-circumscribed deposits of lipids that may be found in skin, tendons, or fasciae [1]. They present as asymptomatic papules, plaques, or nodules [8]. Xanthelasma palpebrarum is the most common presentation of xanthoma, which is the occurrence of xanthoma near the upper eyelids [9]. Xanthelasma is estimated to have a prevalence in the general population of four percent [1]. Cutaneous xanthomas most commonly present in adulthood, with no apparent variance in prevalence between men and women [9]. The pathogenesis of xanthomas is related to the cutaneous deposition of lipids and subsequent inflammation. For xanthomas that occur in the setting of hyperlipidemia, it is thought that the high levels of serum lipoproteins extravasate to the extracellular space [10]. Macrophages are subsequently recruited to consume the lipoproteins and converted to foam cells; these aggregates present clinically as xanthomas [10,11]. Primary hyperlipidemia (i.e., familial hypercholesterolemia) as well as secondary hyperlipidemia (i.e., obesity, diabetes, and hypothyroidism) may present with xanthomas [1,12,13]. The mechanism by which xanthomas occur in the absence of dyslipidemia is not well known. One proposal is that monoclonal gammopathies generate antibodies which cause lipids to accrue in macrophages via immune complexes [14]. Subtypes of xanthomas include planar, eruptive, tuberous, tendinous, and verruciform, with planar xanthomas being the most common [12]. The evaluation of xanthelasma palpebrarum includes the evaluation and correction of any underlying dyslipidemia [1]. Because xanthomas can be a manifestation of highly prevalent cardiovascular conditions such as hypertension and diabetes, it is important to assess the body of information currently available to patients. Furthermore, patients may seek online education on the correlations of this condition with cardiovascular mortality. To our knowledge, there has not been an examination of the quality of online educational materials for xanthelasma palpebrarum. Herein, this study presents a cross-sectional analysis of the quality and content of digital health education on xanthelasma palpebrarum. We aim for this profile of digital information to direct clinicians in improving patient education.

## 2. Materials and Methods

### 2.1. Ethics and Review

This study did not require Institutional Review Board approval as all data utilized in this study is available for public use and did not involve human or animal subjects.

### 2.2. Summary

Inclusion criteria for extracted articles required them to provide pertinent information regarding xanthelasma palpebrarum. This was determined by the screeners after a joint education session on medically accurate and relevant information for xanthelasma. Additionally, articles were evaluated for the following: (1) Written in the English language; (2) Contained over 200 words; (3) Content was publicly available without the need for a content subscription; (4) Content was pertinent to providing information on the search query of interest. Articles were excluded if the associated access link was nonfunctional, a duplicate of a previous link, or did not meet further inclusion criteria [15].

Additionally, frequently asked questions and associated online articles regarding xanthelasma palpebrarum were investigated using publicly available data from May 2023 using the Google RankBrain (Google Inc., Mountain View, CA, USA) machine learning algorithm after the application of inclusion and exclusion criteria. This algorithm was specifically utilized via the search engine function of Google [16,17]. The algorithm provided the most common questions and the associated single article links which Google provides within its search results. These were recorded by the study coordinators.

After questions and articles were extracted, two independent raters evaluated questions for Rothwell’s Classification of Questions. Regarding extracted articles, their content was further reformatted into plain text on Microsoft Word [18,19,20,21,22,23]. Moreover, the irrelevant content material was removed by the screeners (FMQ, SS) from the plain text if the screeners identified that the content was unrelated to education on xanthelasma palpebrarum. This included removal of author information, copyright disclaimers, acknowledgments, references, and any webpage-navigation text. All remaining content remained unchanged when converted to individual plain text documents. Raters then utilized 6 readability scales (i.e., Flesh Reading Ease) and Brief DISCERN (cut-off ≥ 16) for each associated educational article [24,25]. Descriptive statistics for readability calculations were completed using the Stata 14 Statistical Package ^®^ (StataCorp, College Station, TX, USA).

## 3. Results

**Figure 1:**
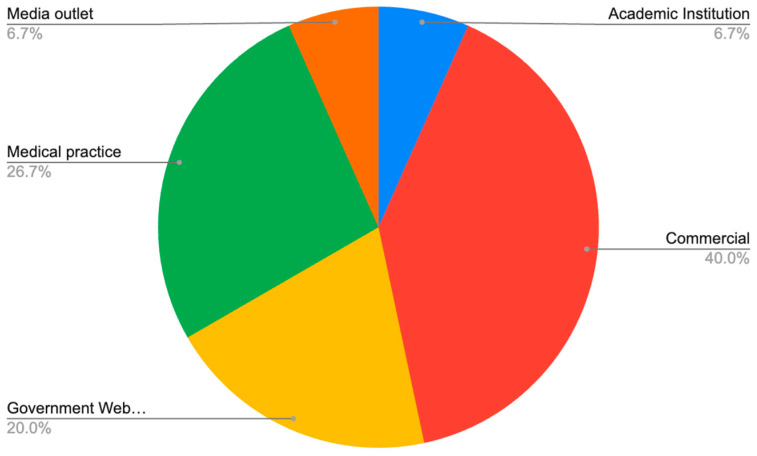
Data Source Classification.

This study analyzed the first thirty unique frequently asked questions and associated online articles regarding xanthelasma palpebrarum. The profile of Rothwell’s Classifications of Questions revealed most questions were considered “Fact” questions at 80.0% (*n* = 24). Upon subclassification, questions regarding “Technical Details” were most common at 70.0% (*n* = 21) followed by questions regarding cost at 6.7% (*n* = 2) and timeline of recovery at 3.3% (*n* = 1). Following this category, questions were next classified as “Policy” at 13.3% (*n* = 4). Upon subclassification of the “Policy” category, all questions pertained to the risks and complications of xanthelasma palpebrarum. The least common classification of questions was “Value” at 6.7% (*n* = 2). Upon subclassification of the “Value” category, questions regarding “Evaluation” and “Pain” were found at 3.3% each (*n* = 1; *n* = 1). The source of questions and articles was most commonly a “Commercial” source at 40% (*n* = 12). This was followed by the source “Medical Practice” at 26.7% (*n* = 8), then “Government Website” at 20.0% (*n* = 6). Furthermore, sources from “Academic Institution” and “Media Outlet” were the least common at 6.7% each (*n* = 2; *n* = 2) (Figure 1). The inter-rater reliability of Rothwell’s Classification of Questions was 90.0%.

**Figure 2:**
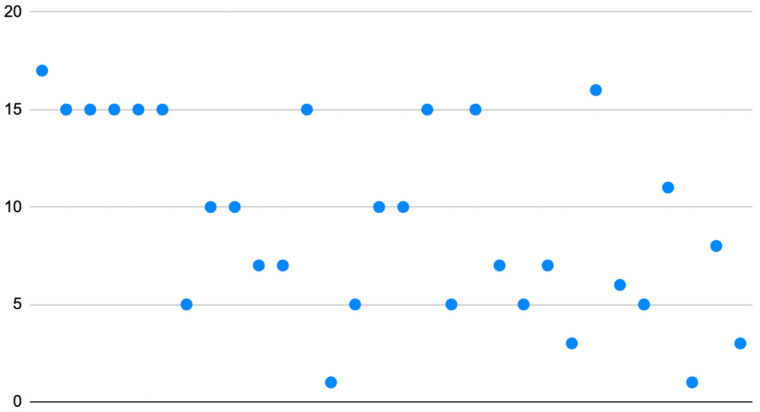
Distribution of Brief DISCERN Scores.

**Figure 3:**
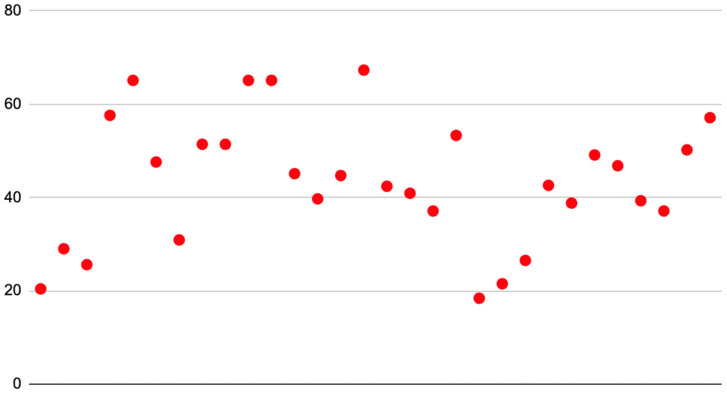
Flesh Reading Ease Score Distribution.

Regarding assessment of the quality of websites, the Brief DISCERN instrument implemented a maximum score of 30, and the average Brief DISCERN of this analysis was 9.3 (SD = 4.9; Range = 1 to 17) (Figure 2). The grade reading level of extracted articles was calculated across six readability formulas. The average Flesh–Kincaid reading score was 11.4 (SD = 3.5). The average Flesch Reading Ease score was 43.6 (SD = 13.7) which is classified as “Difficult to read” or college-level (Figure 3). The average Gunning–Fog score was 13.1 (SD = 2.8). The average Coleman–Liau Index was calculated at 12.3 (SD = 2.4). The average SMOG was 10.3 (SD = 2.8). The average Linsear Write score was 11.1 (SD = 4.9) (Table 1). There was no statistically significant correlation between Flesch Reading Ease Scores and Brief DISCERN scores (*p* = 0.17).

## 4. Discussion

This study aimed to describe the content, readability, and quality of publicly available digital educational resources on xanthelasma palpebrarum [26,27]. Utilization of the internet as a repository of educational resources has allowed for increased dissemination of information [15,26]. However, the paucity of regulatory mechanisms which can observe the complexity and authenticity of these resources has contributed to the results of our study. Specifically, the ability of patients to comprehend these educational resources was evaluated through the implementation of readability calculations. These calculations have been established in multiple research studies and can directly indicate whether educational material meets standard metrics such as the recommended grade reading level in the United States, which is between 6th and 8th grade reading levels [28,29,30]. This reading level can be quantified using the readability calculations implemented in this study. For example, the Gunning–Fog score can be directly translated to a grade category (i.e., a Gunning–Fog score of 8.3 suggests an 8th-grade reading level). This numerical association can be applied to Coleman–Liau Index, SMOG, Linsear–Write, and Flesch–Kincaid calculations. The Flesch Reading Ease score translates to the level of difficulty. Moreover, the Flesch Reading Ease scores are correlated as follows: 0–30 (very difficult), 30–49 (difficult), 50–59 (fairly difficult), 60–69 (standard), 70–79 (fairly easy), 80–89 (easy), and 90–100 (very easy) [31,32,33]. Given the standardized nature of the Flesch Reading Ease score, this study considered the score between 60 and 89 as meeting grade reading level recommendations in the United States, as modeled by the previous literature [15,34]. The findings of this study indicate that the range of mean readability scores was between the 10th to college grade reading levels (SMOG: 10.3, Gunning–Fog: 13.1). Similarly, Figure 2 demonstrates that the distribution of the Flesh Reading Ease score is largely not within the recommendations scores of 60–89. These readability calculations indicate that the currently available patient educational resources on xanthelasma palpebrarum do not meet the recommendations for grade reading levels in the United States but rather are of a higher complexity. This higher complexity may be due to a lack of paraphrasing or simplification of language regarding the subject matter. This high complexity can result in less optimal comprehension by patients regarding xanthelasma palpebrarum [35,36]. A potential modality to improve these patient educational resources may be through transparency of peer review of the articles. Future investigations ought to utilize the readability calculations to further categorize whether there are specific “hotspots” which are more difficult for comprehension regarding xanthelasma palpebrarum (i.e., treatment options and costs).

Additionally, the findings of our readability calculations provide further explanation for the findings of Rothwell’s Classification of Questions in this study. The use of Rothwell’s Classification of Questions was implemented to categorize the frequently asked questions regarding xanthelasma palpebrarum. Specifically, this tool served to understand the specific components of xanthelasma palpebrae that patients’ inquiries focused on. Rothwell’s classification was implemented in previous literature which used internet-based public resources in clinical medicine, but to the best of our knowledge, this has not been utilized for xanthelasma palpebrae before [37,38]. The large majority of questions asked by patients online were categorized as “Fact” based questions (80%), which indicates that the current literature has a concerning paucity of information that can effectively address the questions asked to investigate xanthelasma palpebrarum. The further subclassification of these questions indicates that the technical details of xanthelasma palpebrarum are less known, creating the desire for patients to ask questions to fill this knowledge gap. Likewise, the presence of this knowledge gap may be contributed to by poor readability of the current educational materials on xanthelasma palpebrarum. Further statistical analysis ought to be performed in future studies to investigate this hypothesis.

Brief DISCERN was implemented as the third tool (i.e., readability, Rothwell’s Classification of Questions, and Brief DISCERN) to address the aim of this study. Specifically, this tool has been validated to serve as a threshold marker for quality, evidence-based information. However, this dataset of educational materials of xanthelasma palpebrarum was classified as low quality for most resources, regardless of source (i.e., academic institutions, commercial, government websites, etc.). Only two resources were classified as good quality. Specifically, the Brief DISCERN tools observe deficits in the clarity of references used, addressal of the associated question, and sources of support. This tool categorizes these materials to be of low quality, in addition to the low readability found in this study [24,39,40,41,42,43,44].

To our knowledge, this was the first cross-sectional study that analyzed the quality, readability, and content of patient educational materials of xanthelasma palpebrarum. Given this study is the first in addressing this aim, it is not without its deficits that can be addressed in future studies. For example, this study implemented the use of the RankBrain algorithm. This tool has been utilized in previous literature, and Google’s search engine share of the internet is greater than other engines by over 90% [45,46]. However, this study still did not account for other search engines, which indicates the need for future studies to observe if the findings of the current study are consistent with other search engine systems. Similarly, the use of Rothwell’s Classification of Questions is subjective in nature as a tool, which may suggest potential bias in raters scoring [16,38,47,48,49,50,51]. However, to address this, the high inter-rater reliability of this study addresses the validity of the scoring by the raters.

## 5. Conclusions

Xanthelasma palpebrarum may indicate underlying chronic diseases which significantly contribute to general morbidity and mortality, such as dyslipidemia. Patients with xanthelasma are turning to the internet for education on this condition. This study implemented tools established in the literature to profile the quality, readability, and content of patient educational materials on xanthelasma palpebrarum. The overall profile of these materials is poor and requires dissemination of higher-quality materials. This finding is important for clinicians when aiming to educate their patients on this condition. Moreover, we recommend that clinicians involved in the diagnosis and management of xanthelasma palpebrarum educate their own patients directly in the clinical setting to circumvent the currently poor profile of online educational materials. Thereby, patients may rely on their providers for healthcare literacy as opposed to unreliable online sources. Furthermore, we aim to repeat this analysis for another serious or high-burden disease.

## Figures and Tables

**Table 1:** Average Readability Scores of Digital Resources regarding Xanthelasma Palpebrarum.

	Flesch-Kincaid	Flesch Reading Ease	Gunning-Fog	Coleman-Liau Index	SMOG	Linsear Write
Mean	11.4	42.1	13.1	12.3	10.3	11.1
σ (SD)	3.5	17.7	2.8	2.4	2.8	4.9

## Data Availability

Not applicable.
